# Association between *PLCE1* rs2274223 A > G polymorphism and cancer risk: proof from a meta-analysis

**DOI:** 10.1038/srep07986

**Published:** 2015-01-23

**Authors:** Wenji Xue, Meiling Zhu, Yiwei Wang, Jing He, Leizhen Zheng

**Affiliations:** 1 Department of Oncology, Xin Hua Hospital affiliated To Shanghai Jiaotong University School of Medicine, Shanghai 200092, Shanghai, China; 2 State Key Laboratory of Oncology in South China, Department of Experimental Research, Collaborative Innovation Center for Cancer Medicine, Sun Yat-Sen University Cancer Center, Guangzhou 510060, Guangdong, China

## Abstract

Phospholipase C epsilon 1 (PLCE1) plays an important role in cell growth, differentiation and oncogenesis. An increasing number of individual studies have investigated the association between *PLCE1* rs2274223 polymorphism and cancer risk, but the conclusions are inconclusive. To obtain a comprehensive conclusion, we performed a meta-analysis of 22 studies with 13188 cases and 14666 controls. The pooled results indicated that *PLCE1* rs2274223 A > G polymorphism was associated with an increased risk of overall cancer (G vs. A: OR = 1.15, 95% CI = 1.06–1.25; GG vs. AA: OR = 1.30, 95% CI = 1.10–1.55; GA vs. AA: OR = 1.18, 95% CI = 1.08–1.30; GG/GA vs. AA: OR = 1.20, 95% CI = 1.08–1.32; GG vs. GA/AA: OR = 1.22, 95% CI = 1.04–1.42). The stratification analysis showed the polymorphism was significantly associated with an increased risk of esophageal squamous cell carcinoma (ESCC) other than gastric cancer (GC), especially among the subgroups of Asian, high quality score, sample size > 1000 and the studies consistent with Hardy-Weinberg equilibrium (HWE). This meta-analysis demonstrated that *PLCE1* rs2274223 A > G polymorphism may be associated with increased susceptibility to cancer, especially for ESCC. However, due to the substantial heterogeneities across the studies, the conclusion might be not conclusive that need more studies to confirm.

Cancer is a worldwide problem and its incidence is increasing year by year, which severely endangers the human health and lives. In 2008, the world has more than 12 million new cases of cancer and by 2030, this figure will be over 25 million^1^,^2^. Until now, the pathogenesis of cancer has not been clarified. A majority of studies suggest it may contribute to the cooperation of environmental factors, genetic susceptibility and acquired susceptibility. And, what is noteworthy is that part of the cancer susceptibility comes from human genome diversity^3^.

As genome-wide association studies (GWASs) strategy is putting forward, gene mutations or susceptibility loci have been identified for many diseases. To date, more than 50 GWASs focused on cancer have been published, comprising at least 15 different kinds of malignant tumors^4^. In 2010, Abnet et al firstly performed a GWAS of gastric cancer (GC) and esophageal squamous cell carcinoma (ESCC) in Chinese populations and identified variants located in the *PLCE1* gene at chromosome 10q23 had a genome-wide significantly correlation with gastric cardia cancer (GCA) and ESCC^5^. At the same period, Wang et al also found two susceptibility genes, *PLCE1* and *C20orf54*, were associated with risk of ESCC and GCA for Chinese subjects in another large-scale GWAS^6^. Later, Wu et al further confirmed seven single nucleotide polymorphisms (SNPs) at five regions significantly associated with ESCC, among which is *PLCE1* rs2274223 that has also been found in the former two GWASs^7^.

Rs2274223, located in the 26th exon of the *PLCE1* gene, is a non-synonymous SNP that can cause the amino acid change from histidine to arginine^6^. Since three related GWASs were completed, SNP rs2274223 in *PLCE1* became one of the most studied polymorphic loci. A growing number of studies have been conducted to verify its association with kinds of cancer risk. However, the findings were inconsistent. The discrepancies among these studies might attribute to the relatively small sample size in each research and ethnic variation. Therefore, we performed a meta-analysis of all eligible case-control studies to systematically estimate the effects of *PLCE1* rs2274223 A > G polymorphism on the susceptibility to cancer.

## Results

### Characteristics of eligible publications

**Figure 1:**
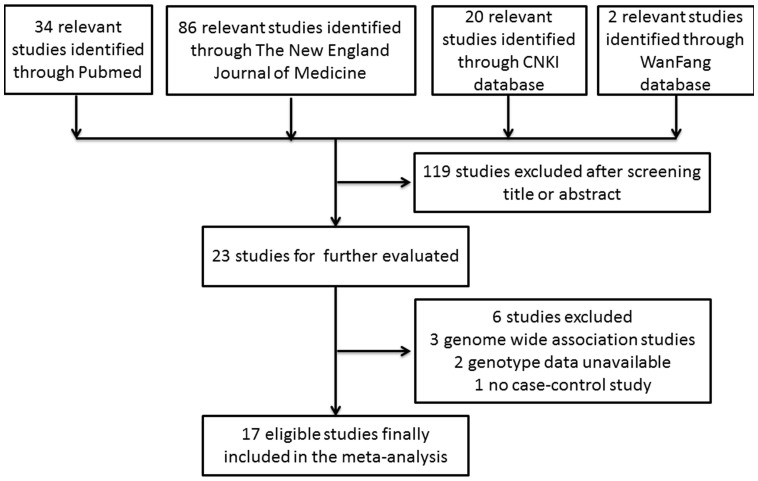
Flow chart of study selection.

A total of 17 eligible articles were identified in the final meta-analysis, which contained 22 studies and involved 13188 cases and 14666 controls. The study selection process was shown in Figure 1^8^,^9^,^10^,^11^,^12^,^13^,^14^,^15^,^16^,^17^,^18^,^19^,^20^,^21^,^22^,^23^,^24^.

Among the 22 studies, six studies reported on GC with 6813 cases and 5666 controls, and nine studies reported on ESCC with 3348 cases and 5309 controls. In the rest of the studies, there were two studies on esophageal adenocarcinoma (EAC), two studies on colorectal cancer, one study on head and neck cancer (HNC), one study on squamous cell carcinoma of head and neck (SCCHN) and one study on gallbladder cancer (GBC). All studies follow the Hardy-Weinberg equilibrium (HWE) except three studies which derived from Palmer's article^15^. In addition, there were eight studies for Caucasian population, 12 studies for Chinese population, one study for African population and one study for mixed population. The main characteristics of those studies are listed in Table 1.

### Results of meta-analysis

**Figure 2:**
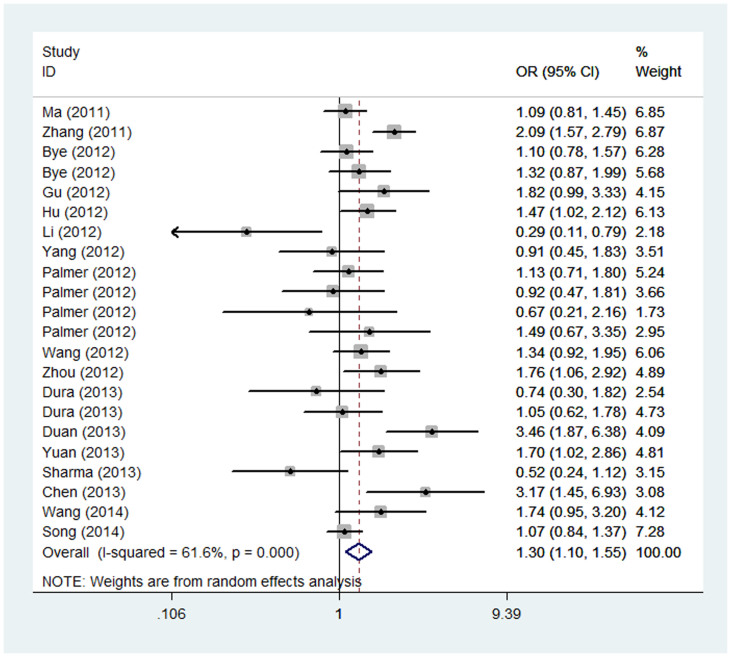
Forest plot of cancer risk associated with *PLCE1* rs2274223 A > G polymorphism (GG vs. AA).

The associations of *PLCE1* rs2274223 polymorphism with the risk of different types of cancer were shown in Table 2, Figure 2. Overall, statistically significant associations were observed among all of the genetic models (G vs. A: OR = 1.15, 95% CI = 1.06–1.25; GG vs. AA: OR = 1.30, 95% CI = 1.10–1.55; GA vs. AA: OR = 1.18, 95% CI = 1.08–1.30; GG/GA vs. AA: OR = 1.20, 95% CI = 1.08–1.32; GG vs. GA/AA: OR = 1.22, 95% CI = 1.04–1.42). Further analysis showed the polymorphism was significantly associated with an increased risk of ESCC other than GC in all genetic models, among which the most obvious is for homozygous model (GG vs. AA: OR = 1.55, 95% CI = 1.17–2.06) and weakest for heterozygous model (GA vs. AA: OR = 1.27, 95% CI = 1.09–1.47).

In GC, stratification analysis by genotyping methods showed that rs2274223 was significantly associated with the risk of GC using TaqMan (G vs. A: OR = 1.20, 95% CI = 1.04–1.39; GA vs. AA: OR = 1.27, 95% CI = 1.12–1.44; GG/GA vs. AA: OR = 1.28, 95% CI = 1.10–1.49). No significant association was detected in the subgroup analysis by ethnicity, source of control, sample size and HWE in controls (Table 3).

In ESCC, significantly increased ESCC risk was only discovered for Asian subgroup (G vs. A: OR = 1.44, 95% CI = 1.25–1.66; GG vs. AA: OR = 2.04, 95% CI = 1.47–2.85; GA vs. AA: OR = 1.39, 95% CI = 1.24–1.56; GG/GA vs. AA: OR = 1.49, 95% CI = 1.29–1.72; GG vs. GA/AA: OR = 1.76, 95% CI = 1.31–2.36), but not for Caucasians and other ethnicities. In the stratified analysis by source of control, significant association was only found for hospital-based controls (G vs. A: OR = 1.29, 95% CI = 1.19–1.41; GG vs. AA: OR = 1.61, 95% CI = 1.29–2.03; GA vs. AA: OR = 1.33, 95% CI = 1.18–1.49; GG/GA vs. AA: OR = 1.37, 95% CI = 1.23–1.53; GG vs. GA/AA: OR = 1.42, 95% CI = 1.13–1.77). When stratified by sample size, we observed a significantly increased risk of ESCC in large sample size whose number was more than 1000 (G vs. A: OR = 1.20, 95% CI = 1.09–1.32; GG vs. AA: OR = 1.34, 95% CI = 1.10–1.64; GA vs. AA: OR = 1.26, 95% CI = 1.12–1.41; GG/GA vs. AA: OR = 1.28, 95% CI = 1.14–1.43). We also performed stratification analysis by quality score, significant association was only identified in high score (G vs. A: OR = 1.22, 95% CI = 1.05–1.41; GG vs. AA: OR = 1.46, 95% CI = 1.10–1.93; GA vs. AA: OR = 1.25, 95% CI = 1.06–1.47; GG/GA vs. AA: OR = 1.27, 95% CI = 1.06–1.52; GG vs. GA/AA: OR = 1.32, 95% CI = 1.05–1.65). Furthermore, in the stratified analysis by HWE in controls, we found a significant increased association between *PLCE1* rs2274223 polymorphism and ESCC risk in the studies consistent with HWE (G vs. A: OR = 1.29, 95% CI = 1.13–1.47; GG vs. AA: OR = 1.62, 95% CI = 1.21–2.15; GA vs. AA: OR = 1.32, 95% CI = 1.19–1.46; GG/GA vs. AA: OR = 1.37, 95% CI = 1.20–1.56; GG vs. GA/AA: OR = 1.42, 95% CI = 1.10–1.83) (Table 3).

### Heterogeneity and sensitivity analysis

We observed substantial heterogeneities among all investigations (GG vs. AA: *P* = 0.000; GA vs. AA: *P* = 0.000; GG/GA vs. AA: *P* = 0.000; GG vs. GA/AA: and G vs. A: *P* = 0.001) (Table 2). The meta-regression analysis yielded no significant difference between subgroup analysis (Supplemental Table 1). We further conducted sensitivity analyses to estimate the influence of each individual data on the combined ORs and no significant differences were observed in all genetic models (Supplemental Figure 1).

### Publication bias

**Figure 3:**
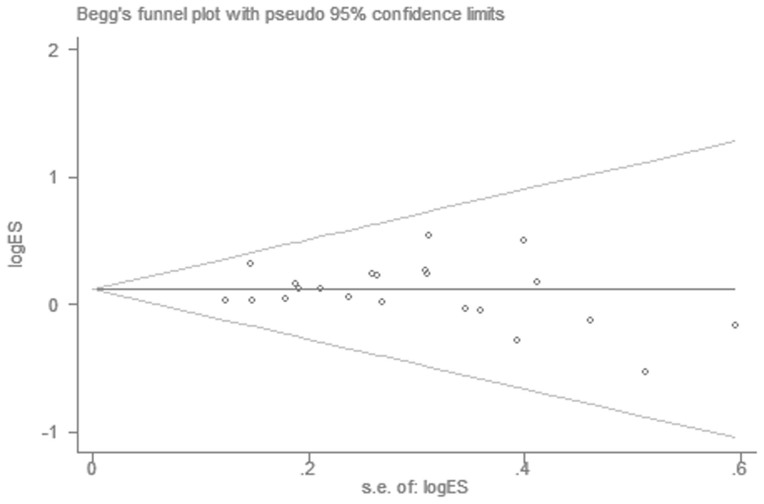
Begg's funnel plot of cancer risk associated with *PLCE1* rs2274223 A > G polymorphism (GG vs. AA).

We performed Begg's funnel plot and Egger's test to assess the publication bias. The shape of the Begg's funnel plot showed basically symmetric distribution. The results of Egger's test were as follows: G vs. A: t = −0.20, *P* = 0.846; GG vs. AA: t = −0.55, *P* = 0.586; GA vs. AA: t = 0.22, *P* = 0.831; GG/GA vs. AA: t = −0.02, *P* = 0.983 and GG vs. GA/AA: t = −0.56, *P* = 0.580, which further provided no evidence of publication bias (Figure 3).

## Discussion

In the meta-analysis, we comprehensively evaluate the association between *PLCE1* rs2274223 polymorphism and cancer risk through 22 studies with 13188 cases and 14666 controls. We observed the genetic variation significantly increased the risk of overall cancer, especially for ESCC. We also found the associations existed in the subgroups of Asian ethnicity, sample size > 1000, high quality score and the studies consistent with HWE in ESCC.

The *PLCE1* gene is located on chromosome 10q23, encoding PLCE1 protein which is a member of phosphoinositide-specific phospholipase C (PLC). PLCE1 protein, like other PLC families, is composed of the PLC catalytic domain, PH domain, EF domain and C2 domain. In addition, PLCE1 protein also has unique regions, two RA domains at its C terminus and a CDC25-like domain at its N terminus. Especially, the former directly interact with several Ras family GTPases, such as oncogenic KRas and HRas. Therefore, PLCE1, as a multifunctional signaling protein, plays an important role in cell growth, differentiation and oncogenesis^25^,^26^,^27^. Simultaneously, an increasing amount of studies start to investigate the association of *PLCE1* rs2274223 polymorphism on the susceptibility of different cancer.

To our knowledge, *PLCE1* mutation is closely associated with many diseases, such as nephrotic syndrome^28^ and cardiac hypertrophy^29^. Although the *PLCE1* rs2274223 polymorphism is associated with a high risk of cancers, the exact mechanism is still unknown. Some studies suggest that PLCE1 protein plays a crucial role in the process of information transmission between the cell membrane and the nucleus. And it probably through augmenting angiogenesis and inflammation, two distinct mechanisms, acts on intestinal tumorigenesis^30^,^31^. Furthermore, the research of gene-gene, gene-environmental interactions may provide some important information. However, the Song's study^32^ indicated that smoking, drinking, and Body Mass Index (BMI) did not significantly change the effect of rs2274223 polymorphism in Chinese population.

Two previously meta-analyses^33^,^34^ have investigated the association between *PLCE1* rs2274223 and different kinds of cancer. Both of them demonstrated that the polymorphism increased the risk of cancer in the pooling analysis, which was the same with ours. In the stratification analysis, the former publication^33^ showed that *PLCE1* rs2274223 polymorphism contributed to the high risk of esophageal and gastric cancer in Asians. However, in the present study, we observed an increased association in Asian ethnicity only for ESCC other than GC. The discrepancy may result from three reasons as the follows: First of all, we classified esophageal cancer into ESCC and EAC for the reason that genetic variation may have different effect on different pathological type of cancer. Because of a limited number of published studies for EAC, we failed to perform further stratification analysis. Therefore, the association between the *PLCE1* rs2274223 polymorphism and EAC risk was ambiguous. Secondly, the positive result about GC might be attributed to small sample size, when we increased the number more than half, we failed to find the significant association. Moreover, the former study mistakenly put the northern Indian population into Asians which may make the result bias. The latter study^34^ identified *PLCE1* rs2274223 polymorphism was associated with the risk of upper aerodigestive tract cancer (ESCC, EC, HNC and SCCHN) but not with gastric and colorectal cancer. However, we did not combine the HNC, SCCHN, EAC and colorectal cancer, because it might be more appropriate to show the results without meta-analysis when the number of studies was less than three.

Compared with the previous publications, our meta-analysis has advantages. Above all, this is by far an analysis with the largest sample size which provided a power of above of 80% investigating the association. What is more, among all the included studies, 95.45% (21/22) were considered as high quality. Furthermore, sensitivity analysis revealed no significant influence of a single study on the summary ORs or the 95% CI. Additionally, no obvious evidence of publication bias existed, which indicated that the result may be unbiased. Some studies have discovered some sequence variants in the region of chromosome, such as 5p15.33 and 8q24, are associated with risk of different cancer types^35^,^36^,^37^,^38^,^39^, so we speculated that rs2274223 may be the specific site associated with different cancer types. However, in the further subgroup analyses, we found rs2274223 significantly associated with an increased risk of ESCC rather than GC which suggested it has no non-specific effect on different types of cancer. So, it appropriate or not to calculate the association of genetic variation with the risk of cancer by pooling the data from different type of cancer remains open to question although researchers have always done this way. Additionally, further functional studies should be carried out to explore the mechanism underlying the variant-related associations with cancer risk.

There was substantial between-study heterogeneity in the meta-analysis. So, although the statistical evidence proved the findings to be reliable, we did not deem lightly of the issue. We used random-effects model to incorporate heterogeneity among studies. However, random-effects model is not a substitute for a thorough investigation of heterogeneity. In addition, we performed the analysis with strict criteria for study inclusion, carried out meta-regression but found no source of heterogeneities. However, further stratification analysis demonstrated that cancer type, source of control and sample size may be the main source of heterogeneities. Besides, there is other heterogeneity that cannot be explained.

Some other limitations should be acknowledged. Firstly, although we had enrolled 22 studies, the sample size for certain cancer types and ethnicities remained relatively small that we could not perform further subgroup analyses. Secondly, we failed to conduct the adjusted estimates for unavailable original data, such as age, sex, smoking and drinking status et al which could make the result masked. Thirdly, some studies are hospital-based design which would not represent the people who live in a certain region. Lastly, even though there is no statistical evidence of publication bias, we may miss some unpublished investigations showing no significant effect or publications written in other languages. Additionally, the power of the funnel plot to test the asymmetry is relatively low when studies were less than 30 or smaller studies were lower methodological quality. What is more, most of the data are retrospective in the meta-analysis. Therefore, reporting publication bias from prospective studies is needed.

In conclusion, the meta-analysis suggests that *PLCE1* rs2274223 polymorphism may be associated with increased susceptibility to cancer, especially for ESCC. However, due to the limitations of the meta-analysis, the conclusion might be not conclusive which need more studies with larger sample size and appropriate design to confirm.

## Methods

### Publication searching

We identified publications from PubMed and The New England Journal of Medicine (OVID platform) using the following search items: “PLCE1” or “Phospholipase C epsilon 1” “SNP” or “polymorphism” or "variant", and “cancer” or "carcinoma" or "neoplasm" or "malignance". We also identified related publications written in Chinese from China National Knowledge Infrastructure (CNKI) and WanFang database using the combinations terms of “PLCE1” or “Phospholipase C epsilon 1” “SNP” or “polymorphism” or "variant", and “cancer” or "carcinoma" in Chinese. The languages were limited to English and Chinese. We further screened the whole references with the date up to April 2014 and confirmed potential relevant studies according to the title and abstract.

### Selection criteria

All studies included in the meta-analysis are accorded with the following inclusion criteria: (a). Full text available; (b). Study focus on the association of *PLCE1* rs2274223 polymorphism and cancer susceptibility; (c). Case-control studies; (d). Genotype data available. In addition, exclusion criteria were as follows: (a). Overlapped articles or repeated studies; (b). Review articles, dissertations and conference reports.

### Data extraction

Two researchers independently extracted data from each eligible study with the following items: the first author, year of publication, the type of cancer, country, ethnicity, source of controls, genotyping technology, number of cases and controls, the quantity of each genotype in cases and controls, Minor Allele Frequency (MAF) and value of HWE. In order to ensure the accuracy of the data, when encountering inconsistent, we discussed together to reach a consensus.

### Quality assessment

According to the quality assessment criteria (Supplemental Table 2), we assessed the quality of each study. Quality scores of studies ranged from 0 (lowest) to 15 (highest). Studies which scores less than 9 were categorized into low quality, while those scores equal to or greater than 9 were regarded as high quality^40^.

### Data analysis

STATA software (version 11.0; Stata Corporation, College Station, TX) was used to perform all analyses. We used allelic (G vs. A), homozygote (GG vs. AA), heterozygote (GA vs. AA), dominant (GG/GA vs. AA) and recessive (GG vs. GA/AA) as the models. The genetic association between the *PLCE1* rs2274223 polymorphism and the risk of cancer was evaluated by the pooled odds ratios (OR) and 95% confidence interval (CI). If the *P* value of the Z test was less than 0.05, we considered the pooled OR statistically significant. Heterogeneity was analyzed using the Chi-squared-based Q-test. When there existed significant heterogeneity (*P* < 0.10), the random-effects model was used^41^. Otherwise, the fixed effects model was used^42^. Stratification and meta-regression analyses were conducted to explore the potential source of heterogeneity across studies. We also performed stratified analyses by cancer type (ESCC and GC), ethnicity (Asian and Caucasian), control source (hospital-based and population-based), quality score of studies (low and high), sample size (>1000 and ≤1000), Genotyping methods (TaqMan and others) and HWE. Sensitivity analyses were performed to measure the stability of the results when one study was removed and the influence of the independent study on the pooled OR at the same time. Publication bias among the literatures was evaluated by Begg's funnel plot and Egger's test (*P* < 0.05 was considered significant)^43^,^44^.

## Author Contributions

L.Z.L. and J.H. conceived and designed this study; Y.W.W., M.L.Z. and W.J.X. searched databases and collected the data; W.J.X. and M.L.Z. performed the statistical analysis, interpretation of data and wrote the manuscript. All authors reviewed the final manuscript.

## Supplementary Material

Supplementary Information Supplementary information

## Figures and Tables

**Table 1:** Characteristics of the 17 publications included in this meta-analysis

Study	Country	Ethnicity	Cancer type	Source of controls	Genotyping methods	Sample sizes(Cases/controls)	HWE (controls)	HWE (cases)	Score
Ma,2011	USA	Caucasian	SCCHN	HB	TaqMan	1097/1089	0.977	0.234	12
Zhang,2011	China	Asian	Gastric	PB	TaqMan	1665/1848	0.451	0.664	14
Bye,2012	South African	African	ESCC	Mixed	TaqMan	418/850	0.886	0.625	11
	South African	Mixed	ESCC	HB	TaqMan	254/857	0.807	0.522	10
Gu,2012	China	Asian	ESCC	HB	MassArray	379/371	0.457	0.653	10
Hu,2012	China	Asian	ESCC	HB	TaqMan	1061/1211	0.577	0.975	12
Li,2012	China	Asian	Colorectal	HB	MassArray	231/292	0.089	0.339	9
Yang,2012	China	Asian	Gastric	HB	MassArray	249/292	0.089	0.539	9
Palmer,2012	Poland	Caucasian	Gastric	PB	TaqMan	289/376	0.307	0.964	10
	USA	Caucasian	Gastric	PB	TaqMan	306/210	0.039	0.036	10
	USA	Caucasian	ESCC	PB	TaqMan	52/210	0.039	0.579	9
	USA	Caucasian	EAC	PB	TaqMan	107/210	0.039	0.834	9
Wang,2012	China	Asian	Gastric	HB	TaqMan	1059/1240	0.224	0.551	11
Zhou,2012	China	Asian	ESCC	HB	PCR-LDR	517/510	0.646	0.318	11
Dura,2013	Netherlands	Caucasian	ESCC	PB	TaqMan	86/580	0.950	0.507	9
	Netherlands	Caucasian	EAC	PB	TaqMan	258/580	0.950	0.554	9
Duan,2013	China	Asian	ESCC	PB	PCR-RFLP	381/420	0.582	0.271	11
Yuan,2013	China	Asian	HNC	HB	TaqMan	501/879	0.243	0.363	12
Sharma,2013	North Indian	Caucasian	GBC	HB	PCR-RFLP	416/225	0.367	0.000	10
Chen,2013	China	Asian	ESCC	HB	MALDI-TOF MS	200/300	0.210	0.895	8
Wang,2014	China	Asian	Colorectal	HB	TaqMan	417/416	0.454	0.953	10
Song,2014	Korean	Asian	Gastric	HB	HRM	3245/1700	0.148	0.088	11

SCCHN, squamous cell carcinoma of head and neck; ESCC, esophageal squamous cell carcinoma; EAC, esophageal adenocarcinoma; HNC, head and neck cancer; GBC, gallbladder cancer; PB, population based; HB, hospital based; HWE, Hardy-Weinberg equilibrium; PCR-LDR, polymerase chain reaction ligase detection reaction; PCR-RFLP, Polymorphism chain reaction-restriction fragment length polymorphism; MALDI-TOF MS, matrix assisted laser desorption ionization/time of flight mass spectrometry; HRM, high-resolution melting.

**Table 2:** Meta-analysis of the association between *PLCE1* rs2274223 A > G polymorphism and cancer risk

Variables	G vs. A			GG vs. AA			GA vs. AA			GG/GA vs. AA			GG vs. GA/AA		
	OR (95% CI)	*P_OR_*^a^	*P*_het_^b^	OR (95% CI)	*P_OR_*^a^	*P*_het_^b^	OR (95% CI)	*P_OR_*^a^	*P*_het_^b^	OR (95% CI)	*P_OR_*^a^	*P*_het_^b^	OR (95% CI)	*P_OR_*^a^	*P*_het_^b^
All	**1.15 (1.06–1.25)**	0.001	0.000	**1.30 (1.10–1.55)**	0.003	0.000	**1.18 (1.08–1.30)**	0.001	0.000	**1.20 (1.08–1.32)**	0.000	0.000	**1.22 (1.04–1.42)**	0.012	0.001
Cancer Type															
GC	1.11 (0.95–1.31)	0.184	0.000	1.26 (0.94–1.68)	0.118	0.010	1.12 (0.92–1.37)	0.253	0.000	1.14 (0.93–1.39)	0.217	0.000	1.22 (0.96–1.54)	0.100	0.057
ESCC	**1.25 (1.08–1.44)**	0.002	0.000	**1.55 (1.17–2.06)**	0.002	0.015	**1.27 (1.09–1.47)**	0.002	0.034	**1.30 (1.10–1.53)**	0.002	0.004	**1.39 (1.10–1.77)**	0.007	0.059
EAC	1.07 (0.88–1.29)	0.503	0.883	1.17 (0.75–1.81)	0.495	0.474	1.05 (0.81–1.37)	0.707	0.511	1.07 (0.83–1.37)	0.601	0.720	1.14 (0.75–1.74)	0.534	0.333
Colorectal	1.02 (0.53–1.95)	0.948	0.001	0.74 (0.13–4.33)	0.742	0.003	1.17 (0.71–1.92)	0.532	0.038	1.10 (0.58–2.10)	0.760	0.005	0.71 (0.15–3.44)	0.668	0.006
HNC	1.14 (0.95–1.38)	0.167	/	1.70 (1.02–2.86)	0.044	/	1.03 (0.81–1.30)	0.807	/	1.09 (0.87–1.37)	0.430	/	1.69 (1.01–2.81)	0.045	/
SCCHN	1.07 (0.94–1.21)	0.291	/	1.09 (0.81–1.45)	0.580	/	1.13 (0.94–1.35)	0.183	/	1.12 (0.95–1.33)	0.188	/	1.02 (0.78–1.35)	0.878	/
GBC	1.09 (0.85–1.40)	0.512	/	0.52 (0.24–1.12)	0.094	/	1.49 (1.07–2.09)	0.020	/	1.35 (0.98–1.88)	0.068	/	0.42 (0.20–0.89)	0.024	/
Publication Bias^c^		0.846			0.586			0.831			0.983			0.580	

^a^*P* value of the Z-test for odds ration test;

^b^*P* value of the Q-test for heterogeneity test;

^c^P value of Egger's test for publication bias; GC, gastric cancer; ESCC, esophageal squamous cell carcinoma; EAC, esophageal adenocarcinoma; SCCHN, squamous cell carcinoma of head and neck; HNC, head and neck cancer; GBC, gallbladder cancer.

**Table 3:** Meta-analysis of the association between *PLCE1* rs2274223 A > G polymorphism and different types of cancer (GC and ESCC) risk

Variables	G vs. A			GG vs. AA			GA vs. AA			GG/GA vs. AA			GG vs. GA/AA		
	OR (95% CI)	*P_OR_*^a^	*P*_het_^b^	OR (95% CI)	*P_OR_*^a^	*P*_het_^b^	OR (95% CI)	*P_OR_*^a^	*P*_het_^b^	OR (95% CI)	*P_OR_*^a^	*P*_het_^b^	OR (95% CI)	*P_OR_*^a^	*P*_het_^b^
GC	1.11 (0.95–1.31)	0.184	0.000	1.26 (0.94–1.68)	0.118	0.010	1.12 (0.92–1.37)	0.253	0.000	1.14 (0.93–1.39)	0.217	0.000	1.22 (0.96–1.54)	0.100	0.057
Ethnicity															
Asian	1.15 (0.94–1.42)	0.180	0.000	1.34 (0.92–1.95)	0.127	0.004	1.15 (0.89–1.48)	0.278	0.000	1.17 (0,90–1.52)	0.228	0.000	1.29 (0.96–1.74)	0.096	0.031
Caucasian	1.03 (0.87–1.22)	0.732	0.409	1.06 (0.72–1.55)	0.772	0.623	1.06 (0.81–1.38)	0.679	0.287	1.05 (0.82–1.35)	0.682	0.293	1.01 (0.70–1.44)	0.967	0.879
Source of control															
PB	1.15 (0,91–1.47)	0.237	0.011	1.37 (0.81–2.33)	0.241	0.019	1.19 (0.97–1.47)	0.097	0.157	1.20 (0.93–1.55)	0.161	0.056	1.29 (0.81–2.06)	0.288	0.030
HB	1.07 (0.88–1.32)	0.487	0.005	1.13 (0.93–1.37)	0.233	0.511	1.09 (0.79–1.50)	0.601	0.000	1.09 (0,81–1.47)	0.561	0.001	1.13 (0.93–1.37)	0.211	0.723
Genotyping methods															
TaqMan	**1.20 (1.04–1.39)**	0.015	0.029	1.39 (0.97–2.00)	0.073	0.033	**1.27 (1.12–1.44)**	0.000	0.254	**1.28 (1.10–1.49)**	0.001	0.122	1.28 (0.92–1.78)	0.135	0.049
MassArray	1.03 (0.77–1.37)	0.848	/	0.91 (0.45–1.83)	0.783	/	1.12 (0.77–1.61)	0.556	/	1.08 (0.76–1.52)	0.668	/	0.87 (0.44–1.74)	0.698	/
HRM	0.96 (0.87–1.05)	0.342	/	1.07 (0.84–1.37)	0.561	/	0.88 (0.77–0.99)	0.034	/	0.90 (0.80–1.01)	0.086	/	1.14 (0.90–1.44)	0.293	/
Sample size															
>1000	1.18 (0.93–1.51)	0.173	0.000	1.44 (0.95–2.20)	0.090	0.002	1.16 (0.86–1.57)	0.333	0.000	1.20 (0.88–1.63)	0.250	0.000	1.37 (0.99–1.89)	0.059	0.025
≤1000	1.03 (0.89–1.19)	0.695	0.711	1.02 (0.73–1.43)	0.902	0.824	1.08 (0.88–1.32)	0.481	0.552	1.06 (0.87–1.29)	0.543	0.572	0.98 (0.71–1.34)	0.887	0.925
HWE															
Yes	1.14 (0.96–1.36)	0.136	0.000	1.30 (0.96–1.78)	0.095	0.007	1.16 (0.93–1,44)	0.190	0.000	1.17 (0.94–1.47)	0.158	0.000	1.24 (0.96–1.60)	0.096	0.040
No	0.95 (0.73–1.23)	0.682	/	0.92 (0.47–1.81)	0.809	/	0.91 (0.63–1.32)	0.629	/	0.91 (0.64–1.31)	0.622	/	0.97 (0.51–1.85)	0.917	/
ESCC	**1.25 (1.08–1.44)**	0.002	0.000	**1.55 (1.17–2.06)**	0.002	0.015	**1.27 (1.09–1.47)**	0.002	0.034	**1.30 (1.10–1.53)**	0.002	0.004	**1.39 (1.10–1.77)**	0.007	0.059
Ethnicity															
Asian	**1.44 (1,25–1.66)**	0.000	0.059	**2.04 (1.47–2.85)**	0.000	0.119	**1.39 (1.24–1.56)**	0.000	0.471	**1.49 (1.29–1.72)**	0.000	0.182	**1.76 (1.31–2.36)**	0.000	0.195
Caucasian	0.82 (0.59–1.13)	0.225	0.264	0.71 (0.35–1.46)	0.354	0.905	0.73 (0.35–1.51)	0.393	0.067	0.73 (0.39–1.37)	0.323	0.097	0.80 (0.40–1.61)	0.539	0.723
African	1.06 (0.89–1.25)	0.521	/	1.10 (0.78–1.57)	0.587	/	1.09 (0.84–1.42)	0.510	/	1.09 (0.85–1.40)	0.474	/	1.05 (0.76–1.43)	0.776	/
Mixed	1.16 (0.95–1.42)	0.139	/	1.32 (0.87–1.99)	0.196	/	1.27 (0.92–1.74)	0.145	/	1.28 (0.95–1.73)	0.109	/	1.14 (0.79–1.65)	0.478	/
Source of control															
PB	1.07 (0.57–2.02)	0.828	0.000	1.28 (0.39–4.14)	0.683	0.004	1.00 (0.50–2.03)	0,992	0.001	1.03 (0.48–2.21)	0.942	0.000	1.33 (0.52–3.38)	0.552	0.027
HB	**1.29 (1.19–1.41)**	0.000	0.496	**1.61 (1.29–2.03)**	0.000	0.365	**1.33 (1.18–1.49)**	0.000	0.950	**1.37 (1.23–1.53)**	0.000	0.829	**1.42 (1.13–1.77)**	0.002	0.338
Mixed	1.06 (0.89–1.25)	0.521	/	1.10 (0.7–1.57)	0.587	/	1.09 (0.84–1.42)	0.510	/	1.09 (0.85–1.40)	0.474	/	1.05 (0.76–1.43)	0.776	/
Genotyping methods															
TaqMan	1.08 (0.93–1.25)	0.300	0.077	1.22 (0.99–1.50)	0.065	0.464	1.09 (0.88–1.35)	0.417	0.065	1.09 (0.88–1.35)	0.420	0.045	1.13 (0.93–1.36)	0.224	0.712
MassArray	1.40 (1.10–1.78)	0.006	/	1.82 (0.99–3.33)	0.052	/	1.42 (1.05–1.94)	0.024	/	1.48 (1.11–1.98)	0.008	/	1.59 (0.88–2.88)	0.124	/
PCR-LDR	1.35 (1.11–1.64)	0.003	/	1.76 (1.06–2.92)	0.029	/	1.39 (1.08–1.80)	0.011	/	1.44 (1.13–1.84)	0.004	/	1.52 (0.93–2.50)	0.096	/
PCR-RFLP	1.86 (1.48–2.35)	0.000	/	3.46 (1.87–6.38)	0.000	/	1.78 (1.31–2.40)	0.000	/	1.97 (1.48–2.62)	0.000	/	2.80 (1.53–5.11)	0.001	/
MALDI-TOF MS	1.54 (1.16–2.05)	0.003	/	3.17 (1.45–6.93)	0.004	/	1.39 (0.95–2.02)	0.087	/	1.55 (1.08–2.22)	0.017	/	2.76 (1.28–5.93)	0.009	/
Sample size															
>1000	**1.20 (1.09–1.32)**	0.000	0.274	**1.34 (1.10–1.64)**	0.004	0.468	**1.26 (1.12–1.41)**	0.000	0.620	**1.28 (1.14–1.43)**	0.000	0.482	**1.20 (1.00–1.45)**	0.050	0.565
≤1000	1.25 (0.92–1.70)	0.146	0.000	1.74 (0.94–3.21)	0.078	0.014	1.21 (0.87–1.69)	0.264	0.006	1.25 (0.87–1.80)	0.220	0.001	**1.67 (1.01–2.75)**	0.046	0.068
Score															
High	**1.22 (1.05–1.41)**	0.011	0.000	**1.46 (1.10–1.93)**	0.008	0.034	**1.25 (1.06–1.47)**	0.008	0.021	**1.27 (1.06–1.52)**	0.009	0.003	**1.32 (1.05–1.65)**	0.017	0.125
Low	1.54 (1.16–2.05)	0.003	/	3.17 (1.45–6.93)	0.004	/	1.39 (0.95–2.02)	0.087	/	1.55 (1.08–2.22)	0.017		2.76 (1.28–5.93)	0.009	/
HWE															
Yes	**1.29 (1.13–1.47)**	0.000	0.003	**1.62 (1.21–2.15)**	0.001	0.016	**1.32 (1.19–1.46)**	0.000	0.367	**1.37 (1.20–1.56)**	0.000	0.071	**1.42 (1.10–1.83)**	0.007	0.040
No	0.66 (0.40–1.07)	0.095	/	0.67 (0.21–2.16)	0.508	/	0.48 (0.25–0.92)	0.028	/	0.51 (0.27–0.94)	0.031	/	0.95 (0.30–2.94)	0.924	/

^a^*P* value of the Z-test for odds ration test;

^b^*P* value of the Q-test for heterogeneity test; GC, gastric cancer; ESCC, esophageal squamous cell carcinoma; PB, Population based; HB, Hospital based; PCR-LDR, polymerase chain reaction ligase detection reaction; PCR-RFLP, Polymorphism chain reaction-restriction fragment length polymorphism; MALDI-TOF MS, matrix assisted laser desorption ionization/time of flight mass spectrometry; HRM, high-resolution melting; HWE, Hardy-Weinberg equilibrium.
