# Supplementary material for: Association between PLCE1 rs2274223 A > G polymorphism and cancer risk: proof from a meta-analysis
Source: Sci Rep. 2015 Jan 23;5:7986. doi: 10.1038/srep07986 (PMC4303865; doi:10.1038/srep07986)
Supplement: Supplementary Information [file srep07986-s1.doc]

**Association** **between** ***PLCE1* rs2274223** **A>G polymorphism and cancer risk: proof from a meta-analysis**

Wenji Xue^1, #^, Meiling Zhu^1, #^, Yiwei Wang^1^, Jing He^2,*^, Leizhen Zheng^1,*^

^1^Department of Oncology, Xin Hua Hospital affiliated To Shanghai Jiaotong University School of Medicine, Shanghai 200092, Shanghai, China.

^2^State Key Laboratory of Oncology in South China, Department of Experimental Research, Collaborative Innovation Center for Cancer Medicine, Sun Yat-Sen University Cancer Center, Guangzhou510060, Guangdong, China.

^#^These authors contributed equally to this work.

**Supplemental Table 1. Meta-regression analyses of the main characteristics of the 22 studies**

| Variable | GG vs. AA | | | GA vs. AA | | | GG/GA vs. AA | | | GG vs. GA/AA | | | G vs. A | | |
| --- | --- | --- | --- | --- | --- | --- | --- | --- | --- | --- | --- | --- | --- | --- | --- |
|  | Coef. | 95% CI | *P* | Coef. | 95% CI | *P* | Coef. | 95% CI | *P* | Coef. | 95% CI | *P* | Coef. | 95% CI | *P* |
| 1 | -0.03 | (-0.12,0.06) | 0.506 | -0.02 | (-0.06,0.03) | 0.448 | -0.02 | (-0.06,0.03) | 0.405 | -0.03 | (-0.12,0.06) | 0.559 | -0.02 | (-0.05,0.02) | 0.366 |
| 2 | 0.01 | (-0.18,0.20) | 0.898 | -0.02 | (-0.14,0.10) | 0.749 | -0.01 | (-0.13,0.10) | 0.840 | 0.01 | (-0.16,0.18) | 0.902 | -0.01 | (-0.09,0.08) | 0.886 |
| 3 | 0.00 | (-0.21,0.21) | 0.997 | -0.02 | (-0.14,0.11) | 0.769 | -0.01 | (-0.13,0.11) | 0.873 | 0.01 | (-0.19,0.20) | 0.939 | -0.01 | (-0.09,0.08) | 0.870 |
| 4 | -0.02 | (-0.08,0.04) | 0.474 | -0.03 | (-0.06,0.01) | 0.100 | -0.03 | (-0.06,0.01) | 0.099 | -0.01 | (-0.07,0.05) | 0.713 | -0.02 | (-0.04,0.01) | 0.140 |
| 5 | -0.05 | (-0.30,0.20) | 0.660 | 0.03 | (-0.10,0.16) | 0.587 | 0.02 | (-0.10,0.15) | 0.692 | -0.07 | (-0.31,0.17) | 0.546 | 0.00 | (-0.10,0.10) | 0.966 |
| 6 | -0.44 | (-1.34,0.47) | 0.321 | -0.10 | (-0.54,0.34) | 0.644 | -0.14 | (-0.56,0.28) | 0.487 | -0.39 | (-1.28,0.49) | 0.357 | -0.15 | (-0.48,0.19) | 0.357 |
| 7 | 0.09 | (-0.50,0.69) | 0.737 | 0.22 | (-0.12,0.56) | 0.182 | 0.21 | (-0.12,0.54) | 0.192 | 0.00 | (-0.56,0.56) | 0.999 | 0.12 | (-0.12,0.37) | 0.300 |

1. Cancer type; 2. Ethnicity; 3. Design; 4. Genotyping method; 5.Sample size; 6. Score;

7. Hardy-Weinberg equilibrium

| Supplemental Table 2. Score of quality assessment | |
| --- | --- |
| **Criteria** | **Score** |
| Representativeness of case |  |
| Selected from population cancer registry | 2 |
| Selected from hospital | 1 |
| No method of selection described | 0 |
| Representativeness of control |  |
| Population-based | 3 |
| Mixed | 2 |
| Hospital-based | 1 |
| Not described | 0 |
| Ascertainment of cancer case |  |
| Histopathologic confirmation | 2 |
| by patient medical record | 1 |
| Not described | 0 |
| Control selection |  |
| Controls matched with cases by age and sex | 2 |
| Controls matched with cases only by age or by sex | 1 |
| Not matched or not described | 0 |
| Genotyping examination |  |
| Genotyping done blindly and quality control | 2 |
| Only genotyping done blindly or quality control | 1 |
| Not described | 0 |
| HWE |  |
| HWE in the control group | 1 |
| HWD in the control group or not mentioned | 0 |
| Total sample size |  |
| > 1000 | 3 |
| 501 - 1000 | 2 |
| 201 - 500 | 1 |
| ≤ 200 | 0 |


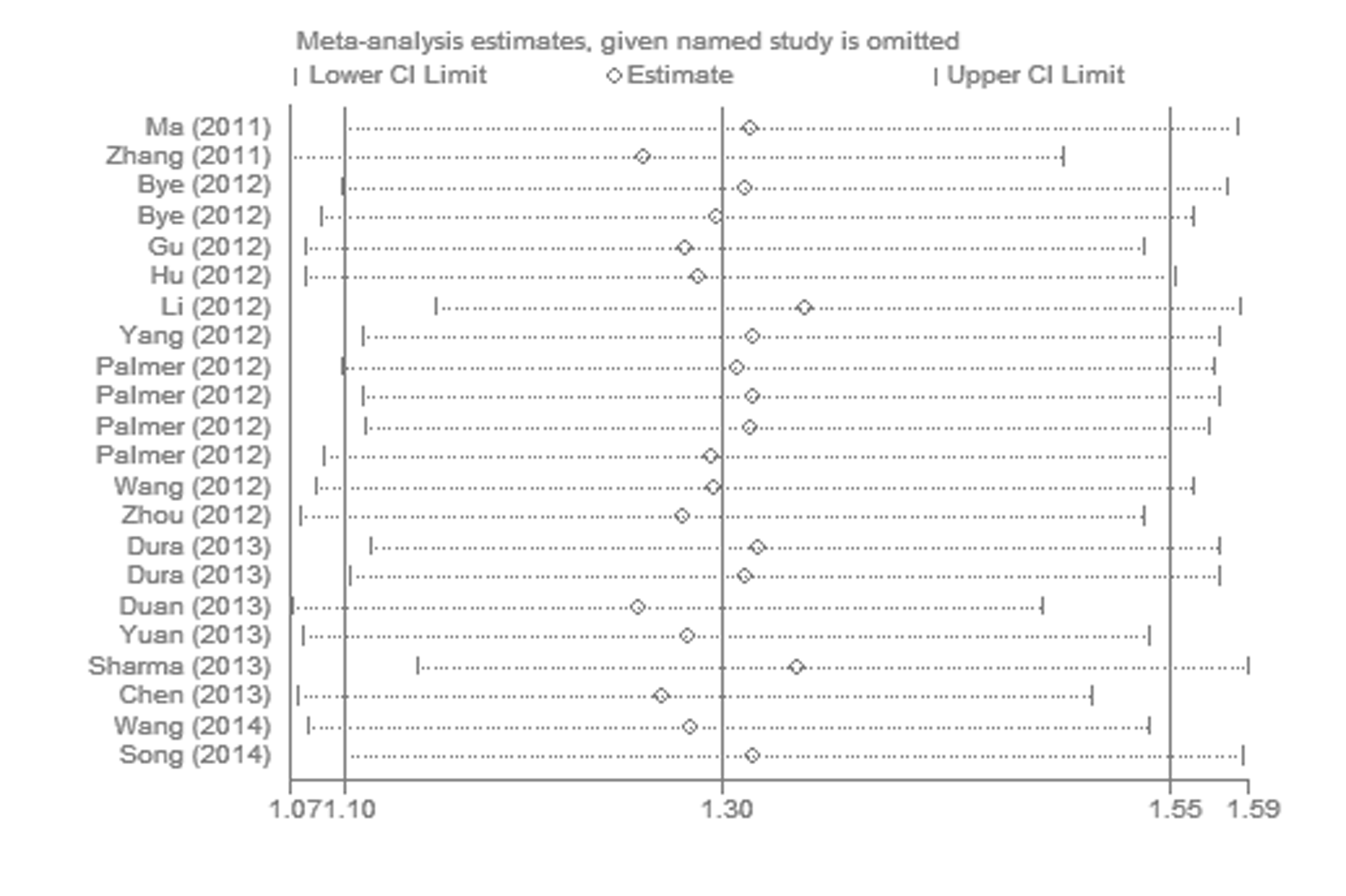


**Supplemental Figure 1.** Sensitivity analysis of cancer risk associated with *PLCE1* rs2274223 A>G polymorphism (GG vs. AA).
